# Monobutyrin and monovalerin improve gut–blood–brain biomarkers and alter gut microbiota composition in high-fat fed apolipoprotein-E-knockout rats

**DOI:** 10.1038/s41598-022-19502-z

**Published:** 2022-09-14

**Authors:** Thao Duy Nguyen, Ayako Watanabe, Stephen Burleigh, Tannaz Ghaffarzadegan, Jirapat Kanklai, Olena Prykhodko, Frida Fåk Hållenius, Margareta Nyman

**Affiliations:** 1 Department of Food Technology, Engineering and Nutrition, Lund University, Lund, Sweden; 2 Laboratory of Nutritional Biochemistry, Department of Applied Biosciences, Graduate School of Bioagricultural Sciences, Nagoya University, Aichi, Japan; 3 Department of Biology, Faculty of Science, Chiang Mai University, Chiang Mai, 50200 Thailand; 4 Present Address: Department of Gastroenterology and Hepatology, Fujita Health University, Aichi, Japan; 5 Present Address: MTM Research Centre, School of Science and Technology, Örebro University, Örebro, Sweden

**Keywords:** Microbiome, Alzheimer's disease, Fatty acids

## Abstract

Monobutyrin (MB) and monovalerin (MV), glycerol esters of short-chain fatty acids (SCFAs), have been shown to positively influence lipid profile and biomarkers in the gut and brain. This study examined whether MB and MV in high-fat diets, affected microbiota composition and gut–blood–brain markers in apolipoprotein E deficient (ApoE-/-) rats, a model for studies of lipid-associated disorders, and neurodegenerative processes in Alzheimer’s disease (AD). ApoE-/- rats fed MB and MV increased *Tenericutes* and the brain neurotransmitter γ-aminobutyric acid (GABA), while the blood stress hormone corticosterone decreased compared to control rats. Only rats that received MB showed a significant increase in cholic acid and *Adlercreutzia* in the caecum. In rats fed MV, the decrease of *Proteobacteria* was associated with decreased corticosterone levels. Conclusively, dietary supplementation of SCFA glycerol esters can modulate gut–blood–brain markers and alter gut microbiota composition in ApoE-/- rats, suggesting that SCFAs also could counteract lipid disorders-related diseases.

## Introduction

There are still no effective treatments for Alzheimer’s disease (AD), the most common neurodegenerative disease in millions of people^1^. In humans, apolipoprotein E allele 4 (*APOE4*) is the major genetic risk factor of AD^2^. ApoE is a cholesterol transporter both in the brain and periphery, with important functions such as controlling neuronal network connectivity, lipid metabolism and systemic inflammation. Individuals carrying the isoform *APOE4* have elevated blood lipids that are associated with increased risk of cardiovascular disease and development of AD^3^. AD patients have impaired brain regions that are controlling stress and memory, as expressed by higher serum levels of the stress marker cortisol^4^ and reduced receptor expression of the neurotransmitter gamma-aminobutyric acid (GABA) compared with healthy controls^5^. In rodents, absence of ApoE mimics similar features seen in humans^6^.

Dietary interventions may represent preventive strategies for combating not only systemic disorders but also neurodegenerative diseases. The reason is the rapid impacts and subsequent physiological changes with different types of diets on gut microbiota composition and their resultant metabolites^7^, that have sparked an increasing interest coined by the term microbiota–gut–brain axis. Short-chain fatty acids (SCFAs) are probably the most studied gut metabolites formed from microbial fermentation of dietary fibres. The three most abundant SCFAs are acetic, propionic, and butyric acids, along with some minor ones including valeric acid^8^. They are related to anti-inflammatory effects, enhanced barrier integrity, and modulated epigenetic and immune response^9^. Upon a high-fat meal, formation of SCFAs is reduced^10^, while levels of bile acids (BAs) are generally increased^11^. Primary BAs are synthesized in the liver and released into the small intestine for facilitating absorption of fats^11,12^. Most BAs are recycled back to the liver; but a small amount, around 5%, escapes to the colon where the microbiota may transform primary BAs to their secondary forms^13^. Primary BAs are considered less toxic to bacterial cells than secondary BAs, exemplified by cholic acid (CA) and its microbially derived counterpart deoxycholic acid^14^. To lower the toxicity of BAs, bacteria can modify BAs to a less toxic form like ursodeoxycholic acid (UDCA). Recent studies also have shed light on the ability of SCFAs on brain functions, especially butyrate, by reducing stress^15^ and improving neurotransmitters related to sleep quality^16,17^. These factors are essential for the balance between normal functions and disease, including AD, of the brain.

Based on our previously obtained data, we hypothesized that glycerol esters of SCFAs could have positive effects on the microbiota profile and biomarkers in the gut (BAs), blood (stress-related marker cortisol in humans or corticosterone in rodents) and brain (neurotransmitter GABA). This idea was investigated in an ApoE knockout (ApoE-/-) rat model, since the ApoE gene is reported to be involved in the development of lipid and brain disorders.

## Materials and methods

### Animals, diets, and experimental design

Conventional (n = 10) and ApoE-/- Sprague–Dawley rats (n = 40), 4–8 weeks old, were used in the study (SAGE Lab, Inc., Boyertown, USA). The rats were adapted to the animal house conditions for 4 days, before entering the 5-week dietary intervention. The ApoE-/- rats were randomly divided into four groups (n = 10/group), where three groups were given high-fat diets and one group a low-fat diet (LF). Two of the high-fat groups were supplemented with monobutyrin (MB) or monovalerin (MV) (10 g/kg diet, dry weight basis), while the third group had no supplementation (HF). The fourth group of ApoE-/- rats was given a diet with low-fat content and no supplementation (LF). Conventional rats (group N) were given the same high-fat diet as the HF group. A detailed description of the experimental design, diets and animals is found in a previous publication^18^. The present experiment was approved by the Ethics Committee for Animal Studies (Review Panel III) at Lund University, Sweden (approval number M114-15). All methods were carried out in accordance with regulations issued by Lund University and the recommendations in the ARRIVE guidelines (Animal Research: Reporting of In Vivo Experiments).

### Analyses

#### Bile acids in caecum

BAs in caecal material were analysed essentially as described by Ghaffarzadegan et al.^19^, with some modifications in the extraction procedure. The caecal contents were freeze-dried using FreeZone Benchtop Freeze dryer (Labconco, MO, USA), and stored at room temperature before analysis. BAs were extracted from 25 mg of milled freeze-dried caecal samples. Ethanol (95%) was added to the samples followed by sonication for 15 min and incubation for 30 min at 60 °C. After cooling in ice for 10 min, the samples were centrifuged at 12,000 g for 10 min at 4 °C. The supernatants were collected and evaporated to dryness and dissolved in 500 µl of ethanol (95%). Then, the solutions were filtered using Minisart® SRP15 syringe filter (Sartorius, Göttingen, Germany) into a GC vial. After evaporating to dryness with a Mivac concentrator (Kovalent, Sweden), the samples were dissolved with 90 µl of derivatization mixture (N-Methyl-N-(trimethylsilyl)-trifluoroacetamide (MSTFA), ammonium iodide (NH4I) and dithioerythritol (DTE); in the ratio (MSTFA:NH4I:DTE (500:4:2, v/w/w)), and 10 µl of internal standard, and then incubated for 30 min at 60 °C. All analyses were performed using an Agilent 6890 N series gas chromatograph (Agilent Technologies, Santa Clara, CA, USA) equipped with an autosampler (Agilent 7683 Series; Agilent Technologies).

#### Microbiota in caecum

DNA extraction of rat caecal samples and generation of 16S rDNA raw sequencing data were performed at Clinical Microbiomics (Copenhagen, Denmark; see Supplementary Materials).

Sequence data were analysed with the open-source bioinformatics pipeline Quantitative Insights Into Microbial Ecology (QIIME)^20^. A total of 2,887,830 reads were obtained from 50 samples with an average of 57,756 reads per sample (min: 37,247 and max: 91,683). The sequences were normalized by rarefaction (depth of 20,000), grouped into operational taxonomic units (OTUs) at a minimum of 97% similarity using the closed reference method based on the Greengenes database (version 13.8) and filtered by the removal of singletons and low abundance OTUs (minimum count fraction set at 0.001). Some QIIME analyses present taxa enclosed in square brackets, which represent taxa similar, but not identical to taxa of the same name without the brackets.

#### Corticosterone in blood

Total concentration of rat serum corticosterone was measured using the DetectX® Corticosterone Immunoassay kit (K014-H1) from Arbor Assays (Michigan, USA), following the manufacturer’s protocol for 5 µl serum sample. Diluted samples and a corticosterone-peroxidase conjugate were pipetted into a clear microtiter plate coated with an antibody to capture sheep antibodies. The binding reaction was started after adding a polyclonal antibody to corticosterone. After incubation and washing the plate, the added substrate reacted with the bound corticosterone-peroxidase conjugate. Then the reaction was stopped, and the intensity of the generated colour was measured in a SpectrostarNano (BMG Labtech) at 450 nm wavelength. For 50 µl of sample, the kit values for sensitivity and detection of limit were 20.9 pg/ml and 17.5 pg/ml, respectively.

#### Gamma-amino butyric acid in the brain

Determination of GABA in brain samples was performed according to Wu et al.^21^ (Supplementary Materials).

### Statistical evaluations

All data were presented as means and standard errors of means (SEM). Data normality was checked by D’Agostino & Pearson test. When comparing all five experimental groups, one-way analysis of variance (ANOVA) was carried out, followed by post-hoc multiple comparisons tests either by Dunnett’s test (for parametric data) or Dunn’s test (for nonparametric data), using the ApoE-/- group fed non-supplemented high-fat diet (HF) as a control. P-values from these multiple comparison tests were corrected and reported as adjusted p values (p_a_). To get a thorough data analysis of a specific group versus the HF group, either 2-tailed unpaired t-test or Mann–Whitney test was used, depending on data normality. A QIIME-based Permanova (using the pseudo-F statistical test and 999 permutations) was used to test for overall differences between treatment microbiomes. Significance level was set at *p* < 0.05, while trends were defined at 0.05 ≤ *p* ≤ 0.1. Multivariate analysis model Partial Least Square-Discriminant Analysis (PLS-DA) was employed to obtain an overview of all data from all experimental groups. To identify specific variables with highest contribution to the separation between two groups, Orthogonal Partial Least Square-Discriminant Analysis (OPLS-DA) was used. These models were validated by cross-validation. Variable Importance for the Projection (VIP) was used to find influencing parameters of group pattern (VIP > 1). Connections of variables were found by Spearman correlation. Univariate and multivariate data analyses were evaluated with GraphPad Prism (version 8) and SIMCA (version 15) software, respectively.

## Results

### Caecal bile acids

There were no major differences in caecal BAs between the two rat models used (groups N and HF) when fed the same type of high-fat diet, an exception was the proportion of β-MCA that tended to be lower in group HF than in group N (p_a_ = 0.0666). Decreasing the amount of fat in diets fed to ApoE-/- rats (LF compared with HF) gave lower amounts of total BAs (Table 1, p_a_ = 0.0797), α-MCA (p_a_ = 0.0742) and the secondary BAs including their sum (DCA, p_a_ = 0.0003; LCA, p_a_ = 0.0043; UDCA, p_a_ = 0.0214; total secondary BAs, p_a_ = 0.0006), while the amount of the primary BA CDCA (p_a_ = 0.012) was higher. Similar results were seen for the proportions of CDCA (p_a_ < 0.0001), DCA (p_a_ = 0.0004), LCA (p_a_ = 0.0027) and UDCA (p_a_ = 0.0856).

**Table 1:** Caecal amounts (mg/caecum) of bile acids (BAs).

	N		HF		MB		MV		LF	
	Mean	SEM	Mean	SEM	Mean	SEM	Mean	SEM	Mean	SEM
**Primary BAs**										
CA (mg/caecum)	3.92	1.29	3.48	0.77	8.80*	2.13	3.70	1.63	3.13	0.62
CDCA (mg/caecum)	1.77	0.60	1.32	0.31	1.43	0.67	1.30	0.35	6.11*	1.19
α-MCA (mg/caecum)	1.62	0.32	1.94	0.32	1.64	0.19	1.49	0.16	1.15†	0.14
β-MCA (mg/caecum)	2.82	0.73	2.77	0.46	2.31	0.32	2.72	0.55	1.75	0.36
Subtotal (mg/caecum)	10.13	2.83	9.51	1.59	14.19	2.55	9.21	2.37	12.14	1.87
**Secondary BAs**										
DCA (mg/caecum)	3.99	1.45	7.35	1.53	5.56	1.82	8.13	1.34	0.01***	0.26
LCA (mg/caecum)	1.24	0.40	2.41	0.56	2.68	1.25	3.22	0.77	0.25**	0.12
UDCA (mg/caecum)	1.12	0.40	1.42	0.32	0.68†	0.19	0.66†	0.20	0.34*	0.08
Subtotal (mg/caecum)	6.36	2.18	11.18	2.20	8.93	3.04	12.02	2.22	0.60***	0.41
*Total BAs* (mg/caecum)	16.49	4.88	20.69	3.53	23.11	4.81	21.23	4.44	12.74†	2.18

Conventional rats (N) fed a high-fat diet and ApoE-/- rats fed either a low-fat diet (LF), a high-fat diet as it is (HF) or supplemented with 1% monobutyrin (MB) or monovalerin (MV). Data are shown as means and their standard errors (SEM). Significant differences compared with the HF group: *, *p* < 0.05; **, *p* < 0.01; ***, *p* < 0.001; †, 0.05 ≤ * p* ≤ 0.1. Cholic acid (CA), chenodeoxycholic acid (CDCA), deoxycholic acid (DCA), lithocholic acid (LCA), ursodeoxycholic acid (UDCA), α-muricholic acid (α-MCA) and β-muricholic acid (β-MCA).

Supplementing MB to the groups fed high-fat diets did not change the total caecal amount of BAs (pool) but altered the composition of BAs by significantly increasing the amount (p_a_ = 0.0297) and proportion (p_a_ = 0.0343) of CA. MB and MV tended to decrease both the total amount (*p* = 0.0642 for MB, *p* = 0.0927 for MV) and proportion (*p* = 0.0433, *p* = 0.0355) of UDCA.

### Caecal microbiota composition

The Shannon index tended to be higher in the MB group compared with the N group (*p* = 0.0727).

#### Phylum level

The nine identified phyla were found in all five groups, where *Firmicutes* (45.3 ± 1.5%), *Bacteroidetes* (33.2 ± 1.5%) and *Verrucomicrobia* (20.5 ± 1.6%) were the most abundant, and there were no significant differences between the groups.

**Figure 1:**
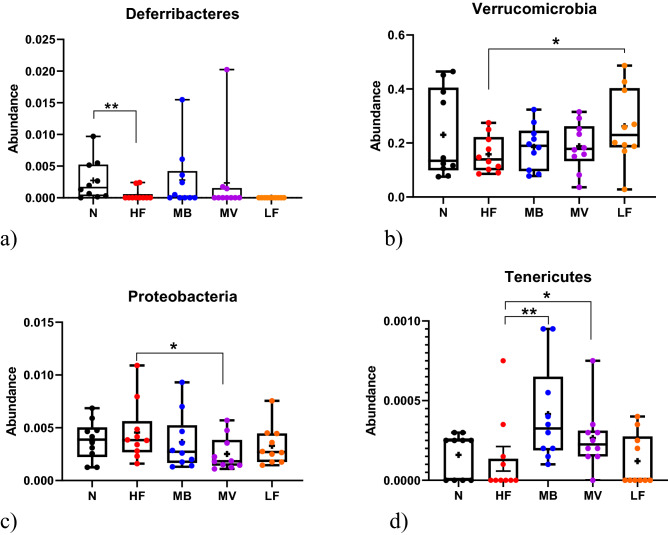
Caecal microbiota at phylum level. (**a**) *Deferribacteres*, (**b**) *Verrucomicrobia*, (**c**) *Proteobacteria* and (**d**) *Tenericutes* in conventional rats (N) fed a high-fat diet and ApoE-/- rats fed either a low-fat diet (LF), a high-fat diet as it is (HF) or supplemented with 1% monobutyrin (MB) or monovalerin (MV). Box and whisker plots show minimum to maximum values, central lines presenting medians and means shown as “ + ”. All data are compared with the HF group. Significant differences compared with this group: *, *p* < 0.05; **, *p* < 0.01.

Conventional rats fed a high-fat diet (N) had more *Deferribacteres* (p_a_ = 0.0162) in caecum than when the corresponding diet fed to ApoE-/- rats (HF) (Fig. 1a). Of the ApoE-/- rats the HF group had less *Verrucomicrobia* (*p* = 0.0482) than the LF group. Supplementing the high-fat diet with MV decreased the amount of *Proteobacteria* (*p* = 0.0355) but tended to possess higher *TM7* (*p* = 0.0872) than the HF group. *Tenericutes* increased in groups fed both MB (*p* = 0.0066) and MV (*p* = 0.0438) compared with HF.

#### Genus level

Of 75 identified genera, 30 were important for the group separation (Supplementary Figure S1a). The five most important bacteria according to the VIP test (in descending order) were *Turicibacter*, *Holdemania*, an unidentified genus in the *Christensenellaceae* family, *Mucispirillum* and *Eubacterium.*

The HF group (Supplementary Figure S1b) had higher abundance of an unidentified genus in the *S24-7* family (p_a_ = 0.0091) compared with the N group, but lower *Parabacteroides* (*p* = 0.0095), *Clostridium* in the *Peptostreptococcaceae* family (*p* = 0.0474), *Streptococcus* (*p* = 0.0266), *Mogibacteriaceae* family (p_a_ = 0.0333), *Holdemania* (p_a_ = 0.0037), *Mucispirillum* (p_a_ = 0.0162), *Dorea* (p_a_ = 0.0218), *Eubacterium* (p_a_ = 0.0657), *Enterobacteriaceae* family (p_a_ = 0.0451), *Ruminococcaceae* family (p_a_ = 0.0054), *Anaerotruncus* (*p* = 0.0759) and *Blautia* (*p* = 0.0524).

Within ApoE-/- groups, the LF group (Supplementary Figure S1c) had lower *Allobaculum* (p_a_ = 0.0182), *Prevotella* (*p* = 0.0332), an unidentified genus in the *S24-7* family (*p* = 0.0224), an unidentified genus of the *RF32* order (*p* = 0.043), an unidentified genus in the *Christensenellaceae* family (p_a_ = 0.0058), and *Sutterella* (*p* = 0.0748) than the HF group, whereas there was higher abundance of *Coprococcus* (p_a_ = 0.0325), *Lactococcus* (p_a_ = 0.014), an unidentified genus (p_a_ = 0.0298) and *Ruminococcus* (p_a_ = 0.054) in the *Ruminococcaceae* family, *Ruminococcus* in the *Lachnospiraceae* family (p_a_ = 0.074), *Streptococcus* (p_a_ = 0.047), an unidentified genus in the *Erysipelotrichaceae* family (*p* = 0.0385), *Holdemania* (*p* = 0.0608), *Enterobacteriaceae* family (p_a_ = 0.0441), *Anaerotruncus* (*p* = 0.0325), and *Akkermansia* (*p* = 0.0482).

Adding MB (Supplementary Figure S1d) to a high-fat diet increased *Adlercreutzia* (p_a_ = 0.0689), *Turicibacter* (*p* = 0.0323), *Clostridia* class (p_a_ = 0.0956), *Coprococcus* (p_a_ = 0.0518), *Tissierella* (*p* = 0.0956), an unidentified genus in the *Erysipelotrichaceae* family (p_a_ = 0.0372), an unidentified genus of the *RF39* order (p_a_ = 0.0235), and *Ruminococcaceae* family (p_a_ = 0.0174) in this group compared with the HF group but decreased *Clostridium* in the *Clostridiaceae* family (*p* = 0.0781).

Supplementing high-fat diets with MV (Supplementary Figure S1e) exhibited higher *Parabacteroides* (*p* = 0.0108), *Turicibacter* (p_a_ = 0.0186), *Coprobacillus* (*p* = 0.0991), *Clostridium* in the *Ruminococcaceae* family (p_a_ = 0.0586), *Anaerotruncus* (*p* = 0.0542), *Mogibacteriaceae* family (*p* = 0.0499), an unidentified genus in the *Erysipelotrichaceae* family (*p* = 0.0313), an unidentified genus in the *F16* family (*p* = 0.0872), and an unidentified genus of the *RF39* order (*p* = 0.0438) in the MV group than the HF group, but gave lower abundance of *Allobaculum* (*p* = 0.0409), *Prevotella* (*p* = 0.068), *Sutterella* (*p* = 0.0657), and *Clostridium* in the *Clostridiaceae* family (p_a_ = 0.0654).

### Serum corticosterone

**Figure 2:**
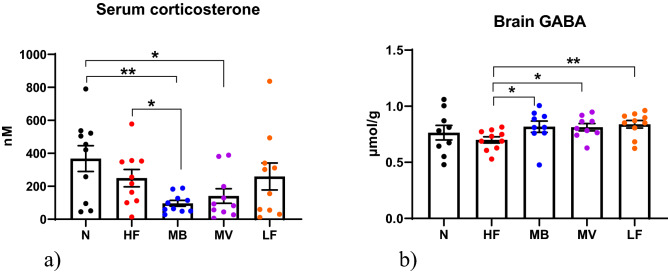
Blood and brain markers. (**a**) Serum corticosterone (nM) and (**b**) brain gamma-aminobutyric acid (GABA, µmol/g) in conventional rats (N) fed a high-fat diet or in ApoE-/- rats fed either a low-fat diet (LF), or a high-fat diet as it is (HF) or supplemented with 1% monobutyrin (MB) or monovalerin (MV). Data are shown as means and their standard errors. Significant differences compared with the HF group: *, *p* < 0.05; ** *p* < 0.01.

Serum corticosterone (Fig. 2a) was significantly lower in the MB group than in the HF (*p* = 0.0123), N (*p* = 0.0033) and LF (*p* = 0.0675) groups. For MV, this level was also lower when compared with the N group (*p* = 0.021) but not with the HF or LF groups.

### GABA in the brain

Brain concentrations of GABA (Fig. 2b) were significantly higher in the LF (0.84 µmol/g, p_a_ = 0.0418), MB (0.82 µmol/g, *p* = 0.0279) and MV (0.81 µmol/g, *p* = 0.0183) groups in comparison with the HF group (0.70 µmol/g). There was no difference in GABA concentration between the N (0.76 µmol/g) and HF group.

### Correlation of caecal microbiota with metabolites

#### Bile acids

**Figure 3:**
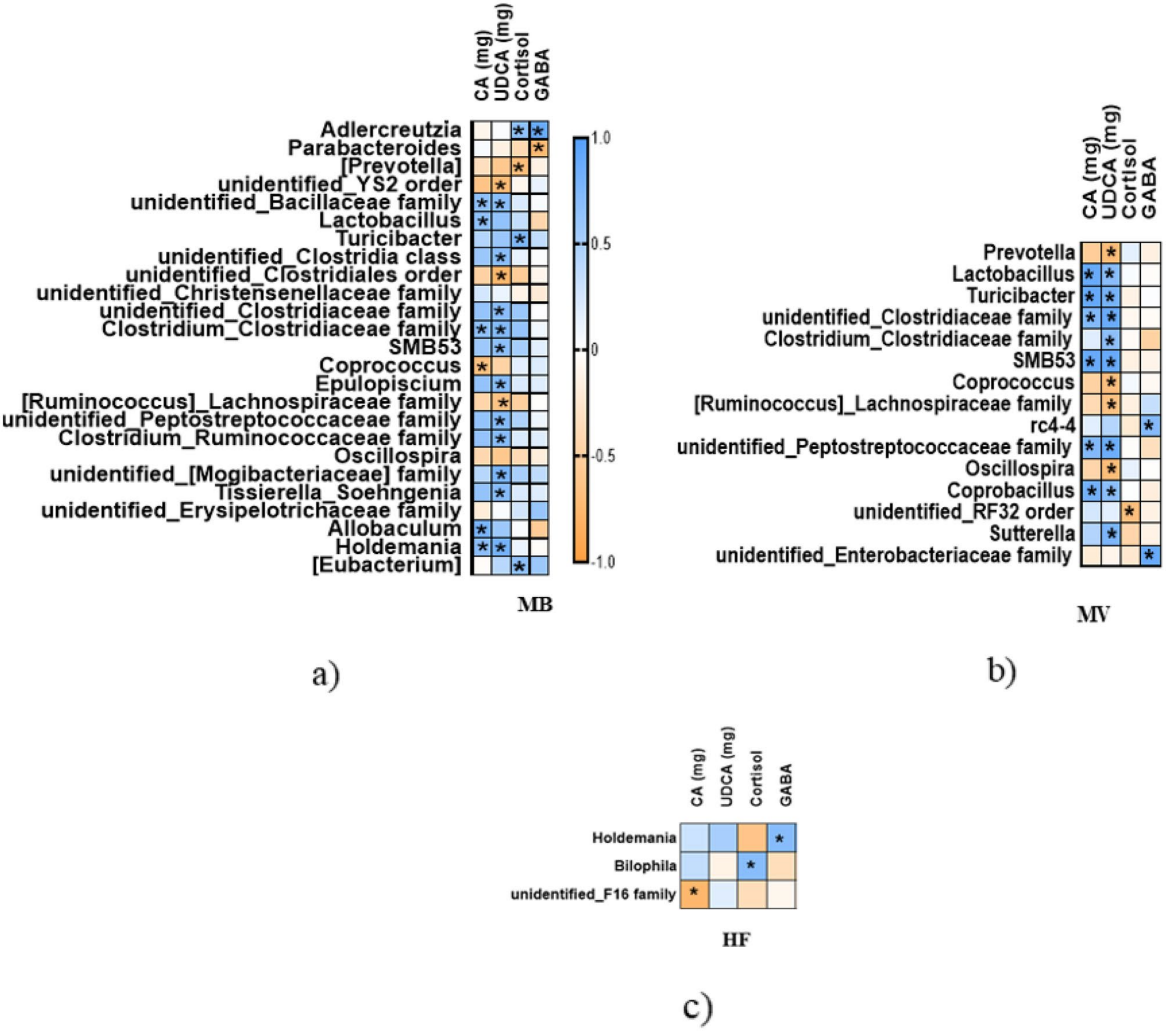
Spearman correlation between caecal bacterial genera and biomarkers. ApoE-/- rats fed a high-fat diet as it is (**c**, HF) or supplemented with 1% monobutyrin (**a**, MB) or monovalerin (**b**, MV). Colour indicator shows positive/negative correlation in blue/orange. Only significant correlations are shown in these figures, which were part of a correlation matrix. Significant values: *, *p* < 0.05.

The amounts of caecal CA and UDCA were associated with the abundance of 17 and 12 bacteria in the MB and MV group, respectively. UDCA was linked to a higher number of bacteria than CA, that can be seen in Fig. 3a,b and more details in the Supplementary materials.

In the HF group (Fig. 3c), only the phylum TM7 and its family *F16* (r = − 0.7622, *p* = 0.0136) were negatively correlated with CA, while UDCA was not connected with any bacteria.

#### Corticosterone

In the MB group (Fig. 3a), corticosterone was positively correlated to *Actinobacteria* phylum and its genus *Adlercreutzia* (r = 0.6512, *p* = 0.0476), *Turicibacter* (r = 0.7509, *p* = 0.0162), and *Eubacterium* (r = 0.653, *p* = 0.0481), and negatively related to *Prevotella* in the *Paraprevotellaceae* family (r = -0.7006, *p* = 0.0222).

In the MV group (Fig. 3b), corticosterone was negatively related to *Proteobacteria* phylum (r = -0.7091, *p* = 0.0268) and its *RF32* order (r = − 0.676, *p* = 0.0373). No associations between corticosterone and bile acids were seen for the MB and MV groups.

In the HF group (Fig. 3c), corticosterone was positively correlated to *Bilophila* (r = 0.6918, *p* = 0.0341), and CA proportion (r = 0.7455, *p* = 0.0174), and negatively correlated to the amounts of CDCA (r = − 0.7697, *p* = 0.0126) and LCA (r = − 0.6848, *p* = 0.0347).

In the N group, corticosterone was found to positively associate with *Blautia* (r = 0.8182, *p* = 0.0058) and negatively correlated with an unidentified genus in the *Christensenellaceae* family (r = − 0.7173, *p* = 0.0237), TM7 phylum and its unidentified genus in the *F16* family (r = − 0.7006, *p* = 0.0304), and CDCA proportion (r = − 0.6485, *p* = 0.049).

#### GABA

In the MB group (Fig. 3a), GABA positively correlated to *Actinobacteria* phylum and its genus *Adlercreutzia* (r = 0.8088, *p* = 0.004), and negatively to *Parabacteroides* (r = − 0.7333, *p* = 0.0311).

In the MV group (Fig. 3b), GABA positively correlated with *rc4-4* (r = 0.7167, *p* = 0.0369), and *Enterobacteriaceae* family (r = 0.887, *p* = 0.0025).

In the HF group, GABA only associated with *Holdemania* (r = 0.7006, *p* = 0.0222).

In the LF group, GABA was positively correlated with α-MCA proportion (r = 0.6727, *p* = 0.039), and negatively with the amount of CDCA (r = − 0.6606, *p* = 0.0438) and *Candidatus Arthromitus* (r = -0.6786, *p* = 0.0289).

### Summary of all data

**Figure 4:**
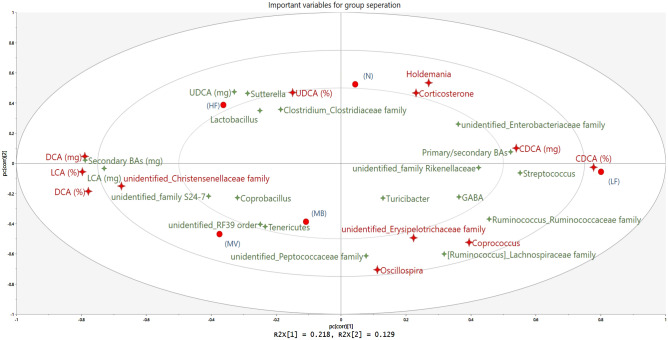
Important variables contributing to group separation. Conventional rats (N) fed a high-fat diet or ApoE-/- rats fed either a low-fat diet (LF), a high-fat diet as it is (HF) or supplemented with 1% monobutyrin (MB) or monovalerin (MV). Groups are shown as red circles, bacteria as 4-point stars. Large red stars are top important variables influencing group separation. Cholic acid (CA), chenodeoxycholic acid (CDCA), deoxycholic acid (DCA), gamma-aminobutyric acid (GABA), lithocholic acid (LCA), ursodeoxycholic acid (UDCA), α-muricholic acid (α-MCA) and β-muricholic acid (β-MCA).

Based on data from all groups, variables important for group separation are presented in Fig. 4. The top 12 variables (marked as red 4-point stars) were CDCA (relative and absolute amounts, % and mg), DCA (% and mg), UDCA (%), LCA (%), *Holdemania*, an unidentified genus in the *Christensenellaceae* family, *Oscillospira*, *Coprococcus*, an unidentified genus in the *Erysipelotrichaceae* family, and corticosterone. The N and HF groups were closely associated with corticosterone and UDCA. The LF group had more CDCA but less DCA, LCA and an unidentified genus in the *Christensenellaceae* family. MB and MV groups were diverged from the N and HF groups, with similar patterns like the LF group for some variables, including lower corticosterone and higher brain GABA, *Coprococcus,* and an unidentified genus in the *Erysipelotrichaceae* family (both belonging to the *Firmicutes* phylum)*.*

## Discussion

In this study we demonstrate that dietary supplementation of glycerol esters of butyric and valeric acids, MB and MV, decreased the stress marker corticosterone in blood and increased the neurotransmitter GABA in the brain of ApoE-/- rats fed high-fat diets. These effects were in parallel with altered compositions of the microbiota and BA profile in the caecum, especially there was a higher amount of CA after supplementation with the glycerol esters. Due to the physiological importance of these investigated parameters and involvement of ApoE in AD, results from this study indicate that dietary supplementation of SCFAs glycerol esters could prevent against disease risk factors associated with lipid disorders.

### Monobutyrin and monovalerin alter the caecal microbiota composition

The caecal microbiota composition of ApoE-/- rats was shifted due to dietary fat content and responded to the presence of the glycerol esters MB and MV. Changes were seen at the phylum level with decreased abundance of *Verrucomicrobia* and its genus *Akkermansia* in the HF group compared with the LF group. This result notes a difference to what is reported in conventional rats where high-fat diets stimulated caecal growth of *Akkermansia*^22^. Further changes between the HF and LF groups occurred at the genus level with some genera in the phyla *Bacteroidetes*, *Firmicutes* and *Proteobacteria*; MB and MV followed mostly the same rhythmic direction of the LF group. A previous study in female ApoE-/- rats reported that *Parabacteroides* (phylum *Bacteroidetes*) and *Eubacterium* (family *Erysipelotrichaceae*, phylum *Firmicutes*) were more abundant in faecal samples after a Western diet (compared with a low-fat diet) at 8 and 20 weeks of age, respectively^23^. In the present study no such changes were seen. Indeed, these two genera were lower in ApoE-/- rats compared with conventional rats fed the same high-fat diets. These differences may be explained by gender, sampling site, age of the rats or experimental time. Our results are very similar with a study in male ApoE-/- mice, where oral gavage of butyrate increased abundance of an unidentified genus (family *Erysipelotrichaceae*) compared with a group fed high-fat diet^24^. This genus was increased not only by the MB group, but also by MV and LF groups in our study.

A group of conventional rats with the same background as the ApoE-/- rats was included in the present study, enabling the comparison on how the same diet may affect microbiota and associated biomarkers in individuals with a knockout gene. *Deferribacteres* and its genus *Mucispirillum* were significantly higher in the N than the HF group. All bacteria that were different between the two groups were mainly higher in the N group than in the HF group, except *S24-7* family which was lower in the N group. The family *S24-7* may have proinflammatory and pathogenic effects as they were elevated in mice colonized with the microbiota of high-cholesterol human donors^25^ and in mice with AD or diabetes^26,27^. It should be noted that MB and MV decreased the abundance of *S24-7* family in conventional rats fed high-fat diets compared with those fed the same diets without supplementation^10,28^. MV also stimulated *Parabacteroides* and *Mogibacteriaceae* family as the N group. Overall MB and MV modulated the HF-induced microbiota composition to a profile like the LF.

### Monobutyrin increases cholic acid

In this study we found MB significantly stimulated the amount of CA in the caecum in comparison with the HF diet. A similar increase in caecal CA was also seen in rats fed high-fat diets enriched with dietary fibres (guar gum and barley β-glucans)^11,29^, accompanied with increased concentrations of caecal SCFAs (all 3 main SCFAs acetic-, propionic-, and butyric acids)^22^. The cholesterol-lowering effect of guar gum (7.5%) was previously reported to be enhanced by greater faecal excretion of BAs in cholesterol-fed rats despite effective reabsorption of BAs in the caecum as well as in the small intestine^30^. There is no precisely direct mechanism between BAs and SCFAs, though they relate to the microbiota. It is known that high-fat feeding generally stimulates BA secretion, while SCFAs formation is depressed. In our previous study with conventional rats, hepatic mRNA expression of *Cyp8b1* was decreased after adding MB to high-fat diets. This enzyme is responsible for the synthesis of CA in the liver, and its hepatic expression was downregulated through the FXR/SHP (farnesoid X receptor/small heterodimer partner or *Nrb02*) pathway^31^. However, MB did not change hepatic expression of *Nrb02*^10^. Other associations with FXR, such as intestinal GPR43 mRNA expression and intestinal permeability were not affected in ApoE-/- rats fed MB^18,32^. It is potentially so that MB might preferentially act towards reduced small intestinal reabsorption of BAs. As shown in a study of ApoE-/- mice, gut microbiota-dependant butyrate was responsible for the improved serum lipid levels with pectin (20% by weight of a high-fat diet) by inhibiting absorption and promoting excretion of cholesterol in the small intestine through increased mRNA expression of liver X receptor alpha (*Lxrα*)^33^. Another study on ApoE-/- mice fed high-fat diet showed that oral gavage of butyrate (400 mg/kg) also promoted reverse cholesterol transport by upregulating ATP-binding cassette sub-family A member 1 (ABCA1) expression in liver, macrophages in lesions of aortic roots and peritoneal macrophages^24^. The increase of CA in this study, complementing our previous data, corroborates protective effects of MB on lipid disorders by increasing BA excretion to the caecum, limiting the return of primary BAs to the liver. To compensate this loss, more cholesterol from the periphery is recruited for hepatic BA synthesis, eventually reducing serum^34^ and liver^10^ cholesterol levels. Therefore, the increased excretion of BAs by MB is reasonable as a molecular mechanism explaining the improved lipid profile.

### Monobutyrin and monovalerin reduce stress markers

An interesting result in this study is that supplementation of MB or MV decreased serum levels of the stress hormone corticosterone in ApoE-/- rats consuming high-fat diets. Increased circulating corticosterone levels have been shown to accelerate dyslipidaemia and atherosclerosis, in parallel with cognitive decline in ApoE-/- mice^35,36^. In healthy mice, 1-week oral administration of a mixture of three main SCFAs (132.5 mmol, mixed in drinking water) was effective in lowering corticosterone levels in plasma sampled by tail-tip at 30 min after an acute stressor^37^. In healthy men, 1-week daily ingestion of a SCFA mixture both at high (239.9 mmol) and low (119.9 mmol) doses was able to attenuate salivary cortisol response to acute psychosocial stress up to 65 min. This effect was associated with increased serum SCFAs measured within 5 h upon waking^15^. In healthy or non-demented elderly, a relationship between higher salivary cortisol levels and worse memory performance has been reported, especially in those carrying the main risk factor of late onset of AD allele ApoE4, suggesting that they are more vulnerable to cognition decline during aging^38,39^. Corticosterone level is also associated with the gut microbiota due to a bidirectional interaction between the hypothalamic–pituitary–adrenal (HPA) axis and gut microbiota. For example, higher plasma corticosterone levels in response to a mild constraint stress in germ-free (lacking a normal microbiota) mice were observed compared with control mice with a normal microbiota composition without specific pathogens^40^. The exaggerated stress response was reversed by mono-association with specific bacterial strains. Cortisol, in return, can change gut microbiota composition, as well as gut permeability and function^41^. The decreased serum corticosterone by MB and MV was correlated with changes in specific bacteria. For instance, the decrease in corticosterone by MB was associated with increased abundances of *Adlercreutzia* (*Actinobacteria* phylum) and *Turicibacter* (*Firmicutes* phylum). *Adlercreutzia* was seen to decrease in faecal microbiota from patients with multiple sclerosis^42^. High levels of this genus can be considered as positive since it may have an anti-inflammatory role and can convert phytoestrogens to monomers, plant molecules naturally found in whole grains, legumes, fruits, and vegetables^43^. Decreased *Turicibacter* was found in the caecum of socially stressed mice and individuals with autism^44,45^. In MV, decreased corticosterone was in parallel with decreased abundance of *Proteobacteria*. This phylum is increased in faecal samples of mice fed high-fat diets and patients with major depressive disorder^46,47^. It seems that MB and MV modulate the presence of some bacteria associated with high-fat feeding, inflammation, and brain disorders. Despite some differences in experimental settings, results from ours and others’ studies point to the same direction that delivering butyrate, through approaches attaching to the oral-gastrointestinal routes, may result in lower levels of the stress marker cortisol or corticosterone, supporting SCFAs roles in the microbiota–gut–brain axis.

### Monobutyrin and monovalerin stimulate neurotransmitters

In contrast to the decrease in serum corticosterone, concentrations of GABA in the brain were increased with both MB and MV. There is evidence of relationship between gut microbiota, GABA, and corticosterone. For examples, oral administration of a *Lactobacillus* strain has been shown to increase brain concentrations of GABA^48^, along with decreased plasma corticosterone levels at 30 min after forced swim test in healthy mice^49^. The increased level of brain GABA was only significantly evident at 4 weeks. Interestingly, MB and MV showed similar effects on blood corticosterone and brain GABA in high-fat fed ApoE-/- rats as did the previous probiotic study by Janik et al*.*^48^. Moreover, the genus *Adlercreutzia* appeared to be increased with increased brain GABA and decreased serum corticosterone in the MB group. Increases in *Adlercreutzia* and butyric acid were seen in faeces of mice who consumed milk fermented with a GABA-producing *Lactobacillus* strain, improving sleep^17^. Taken together, MB can improve beneficial effects, and decrease the opposites in ApoE-/- rats fed high-fat diets, possibly via the blood–brain-barrier by stimulating butyric acid levels and mRNA expression of tight junction proteins (such as occludin) in the brain as shown previously in rats^18^.

To conclude, SCFA glycerol esters as an added dietary component are impactful in modulating adverse effects induced by high-fat regimen and ApoE deficiency on factors critical for gut–brain communication. Specifically, stress-induced corticosterone was reduced in blood and GABA neurotransmitter increased in brain tissue. These outcomes were linked with compositional changes of bacterial community and BA profile in caecum. Dietary intervention with SCFA glycerol esters is thus suggested as preventive strategy to early combat ApoE-related diseases like AD.

Taken together, the present study and our previous findings support the concept that dietary supplementation of SCFA glycerol esters as an effective means to counteract harmful effects of high-fat diets in both normal and ApoE-deficient rats. Positive effects were identified in a wide range of different locations/organs/tissues across the gut–brain axis regarding lipid disorders, barrier permeability, stress, neurotransmitter impairment, and gut dysbiosis. Notably, SCFA glycerol esters showed more pronounced effects in a dose–response manner. Particularly for ApoE-/- rats, the most prominent effects were seen in the brain than in the gut. Thus, higher doses can be tested safely to achieve specific outcomes in future studies, based on the non-toxic maximal dose of 200 mg/kg in humans. These results open the possibility for SCFA glycerol esters to be used as promising dietary components for the prevention of metabolic and neurodegenerative diseases.

## Supplementary Information

Supplementary Information.

## Data Availability

The datasets used and/or analysed during the current study available from the corresponding author on reasonable request. The microbiome and associated biomarker data is available through the Sequence Read Archive (SRA) at the National Center for Biotechnology Information (NCBI). The BioProject accession number is PRJNA871087.

## Abbreviations

AD: Alzheimer’s disease
ApoE: Apolipoprotein E
ANOVA: Analysis of variance
ABCA1: ATP-binding cassette sub-family A member 1
BAs: Bile acids
CA: Cholic acid
CDCA: Chenodeoxycholic acid
DCA: Deoxycholic acid
DTE: Dithioerythritol
FXR/SHP: Farnesoid X receptor/small heterodimer partner
GABA: Gamma-aminobutyric acid
GPR43: G protein-coupled receptor 43
HPA: Hypothalamic–pituitary–adrenal axis
LCA: Lithocholic acid
Lxrα: Liver X receptor alpha
MB: Monobutyrin
MV: Monovalerin
MSTFA: N-Methyl-N-(trimethylsilyl)-trifluoroacetamide
NH4I: Ammonium iodide
OPLS-DA: Orthogonal partial least square-discriminant analysis
OTUs: Operational taxonomic units
PLS-DA: Partial least square-discriminant analysis
QIIME: Quantitative insights into microbial ecology
SCFAs: Short-chain fatty acids
SEM: Standard errors of means
UDCA: Ursodeoxycholic acid
VIP: Variable importance for the projection
α-MCA: α-Muricholic acid
β-MCA: β-Muricholic acid
