# Supplementary material for: Monobutyrin and monovalerin improve gut–blood–brain biomarkers and alter gut microbiota composition in high-fat fed apolipoprotein-E-knockout rats
Source: Sci Rep. 2022 Sep 14;12:15454. doi: 10.1038/s41598-022-19502-z (PMC9475028; doi:10.1038/s41598-022-19502-z)
Supplement: Supplementary file 1 — Supplementary Information. [file 41598_2022_19502_MOESM1_ESM.docx]

**Monobutyrin and monovalerin improve gut-blood-brain biomarkers and alter gut microbiota composition in high-fat fed apolipoprotein-E-knockout rats**

**Thao Duy Nguyen^1^*, Ayako Watanabe^2,4^, Stephen Burleigh^1^, Tannaz Ghaffarzadegan^1,5^, Jirapat Kanklai^3^, Olena Prykhodko^1^, Frida Fåk Hållenius^1^ and Margareta Nyman^1^**

^1^ Department of Food Technology, Engineering and Nutrition, Lund University, Lund, Sweden

^2^ Laboratory of Nutritional Biochemistry, Department of Applied Biosciences, Graduate School of Bioagricultural Sciences, Nagoya University, Aichi, Japan

^3^ Department of Biology, Faculty of Science, Chiang Mai University, Chiang Mai 50200, Thailand

^4^ Current affiliation: Department of Gastroenterology and Hepatology, Fujita Health University, Aichi, Japan

^5^ Current affiliation: MTM Research Centre, School of Science and Technology, Örebro University, Örebro, Sweden

* Corresponding author: Thao Duy Nguyen (Email: thao_duy.nguyen@food.lth.se)

**Supplementary materials**

**Methods**

***GABA analysis***

An amount of 0.2 g rat brain tissue was cut on dry ice and transferred to a homogenization tube. Chilled NaCl solution 0.9% (w/v) was added to the tube (ratio 1:3 (w/v)). Samples were homogenized by hand and centrifuged at 12,000 rpm for 20 minutes at 4°C. The supernatants were collected and kept in the freezer before analysis.

A volume of 100 μl GABA standard solution or sample supernatant were mixed with 175 μl borate buffer solution, 200 μl acetonitrile and 25 μl of NBD-F (4-fluoro-7-nitrobenzofurazan) solution in a centrifuge 1.5 ml tube. The reaction was performed at 60°C in water bath for 7 minutes in dark. After the reaction, the sample tubes were cooled at ambient temperature and then filtrated through 0.2 μm nylon membrane filter, before HPLC analysis.

GABA analysis was performed by using Shimadzu HPLC system (LC-20A, Japan). A volume of 10 μl of GABA standard or sample was separated by reverse-phase (C-18) HPLC analytical column, (ultrasphere octadecylsilyl (ODS) 4.6 mm × 250 mm, 5 μm). Mobile phase was a mixture of acetonitrile and phosphate buffer (0.02 mol/L, pH 6.0) in a ratio of 16:84 v/v. The HPLC analysis was performed isocratic, and a run lasted for 20 minutes with following conditions: flow rate 1.0 ml/min and controlled column temperature 30°C. All peaks were detected by UV detector at 472 nm.

***Microbiota analysis***

DNA was extracted from ~50 mg of rat caecum samples using NucleoSpin® 96 Soil (Macherey-Nagel). Bead beating was done on a Vortex-Genie 2 horizontally at 2700 rpm for 5 min. A minimum of one positive control (ZymoBIOMICS™ Microbial Community Standard, Zymo Research) and four negative controls were included. PCR was done with the forward primer S-D-Bact-0341-b-S-17 (5'-TCGTCGGCAGCGTCAGATGTGTATAAGAGACAGCCTACGGGNGGCWGCAG-3') and reverse primer (5'-GTCTCGTGGGCTCGGAGATGTGTATAAGAGACAGGACTACHVGGGTATCTAATCC-3') S-D-Bact-0785-a-A-21 (Klindworth et al. 2013)^1^ with Illumina adapters attached. These are universal bacterial 16S rDNA primers, which target the V3-V4 region. The following PCR program was used: 98 °C for 30 sec, 25x (98 °C for 10 s, 55 °C for 20 s, 72 °C for 20 s), 72 °C for 5 min. Amplification was verified by running the products on an agarose gel. Indices were added in a subsequent PCR using the Nextera Index Kit V2 (Illumina) with the following PCR program: 98 °C for 30 sec, 8x (98 °C for 10 s, 55 °C for 20 s, 72 °C for 20 s), 72 °C for 5 min. Attachment of indices was verified by running the products on an agarose gel.

Products from the nested PCR were pooled based on band intensity and the resulting library cleaned with magnetic beads. The DNA concentration of pooled libraries was measured fluorometrically. Sequencing was done on an Illumina MiSeq desktop sequencer using the MiSeq Reagent Kit V3 (Illumina) for 2 x 300 bp paired-end sequencing.

**Results**

**Caecal microbiota composition**

*Genus level*


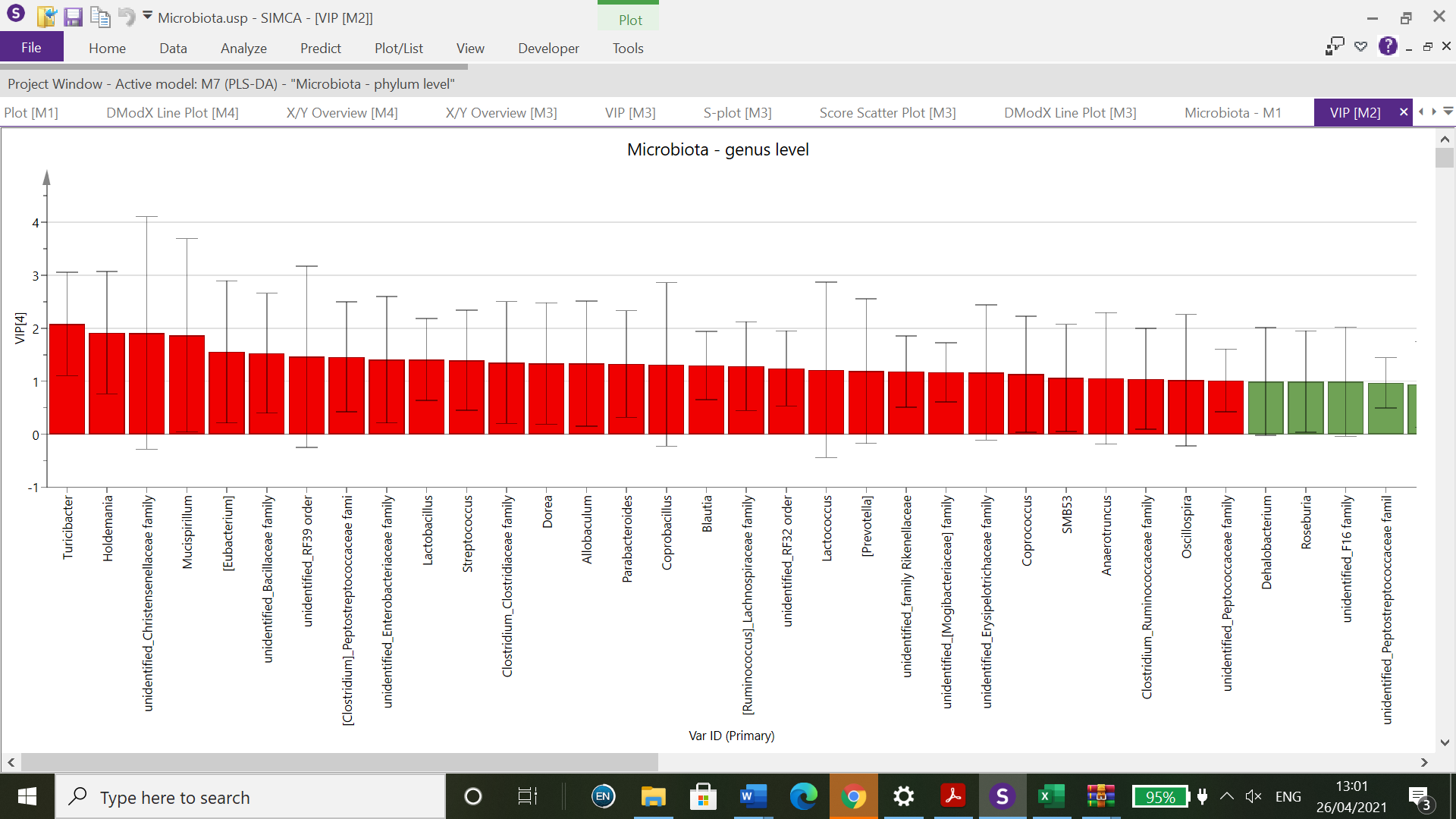


**Figure S1a.** Caecal microbiota at genus level in conventional rats (N) fed high-fat diet or in ApoE-/- rats fed either low-fat diet (LF), a similar high-fat diet as with normal rats (HF) or supplemented with monobutyrin (MB) or monovalerin (MV). Genera marked in red are important for the group separation.


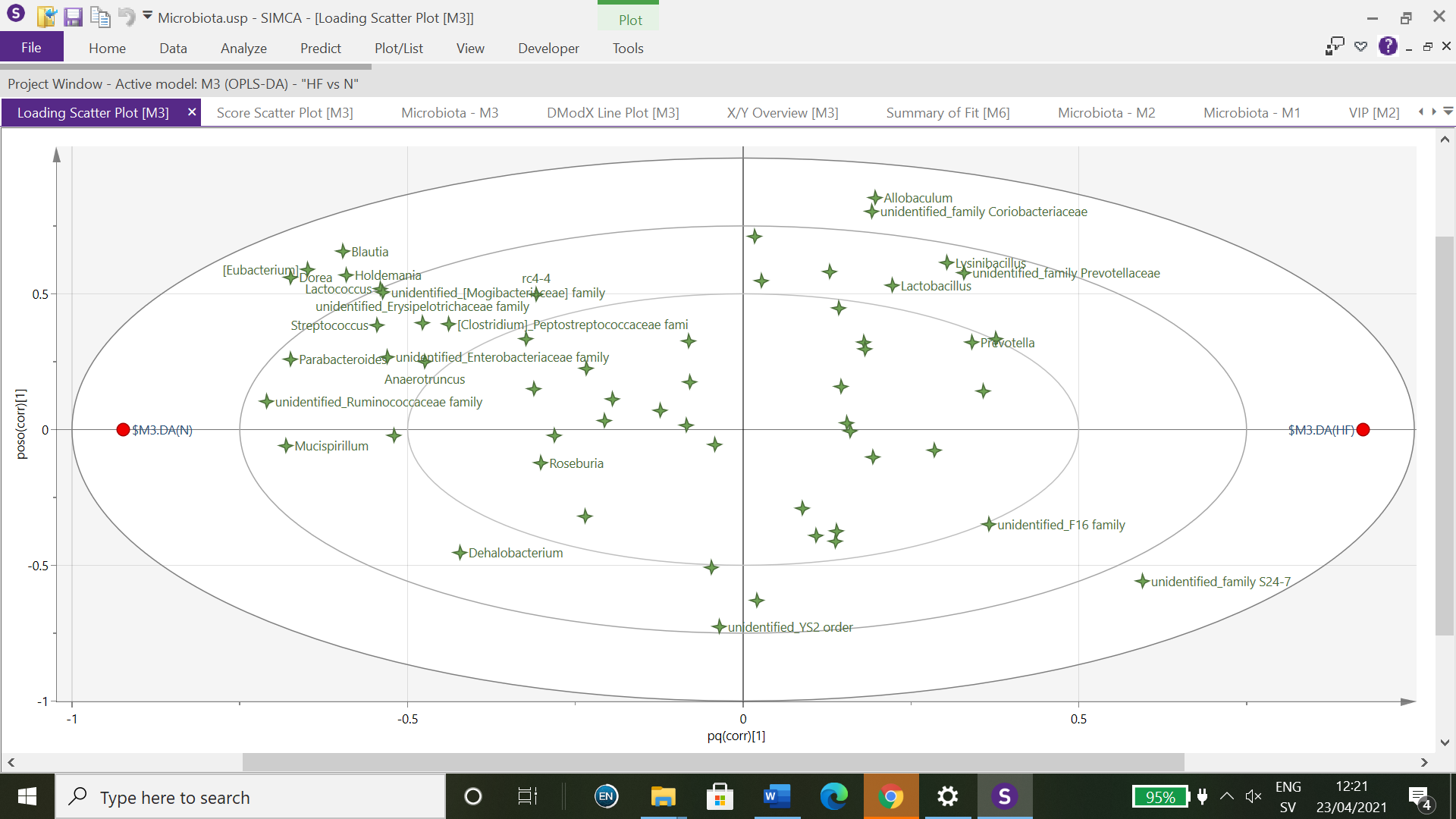


**Figure S1b.** Caecal microbiota at genus level in conventional rats (N) and in ApoE-/- rats (HF) fed high-fat diets. The two groups are shown as circles, bacterial genera as 4-point stars.


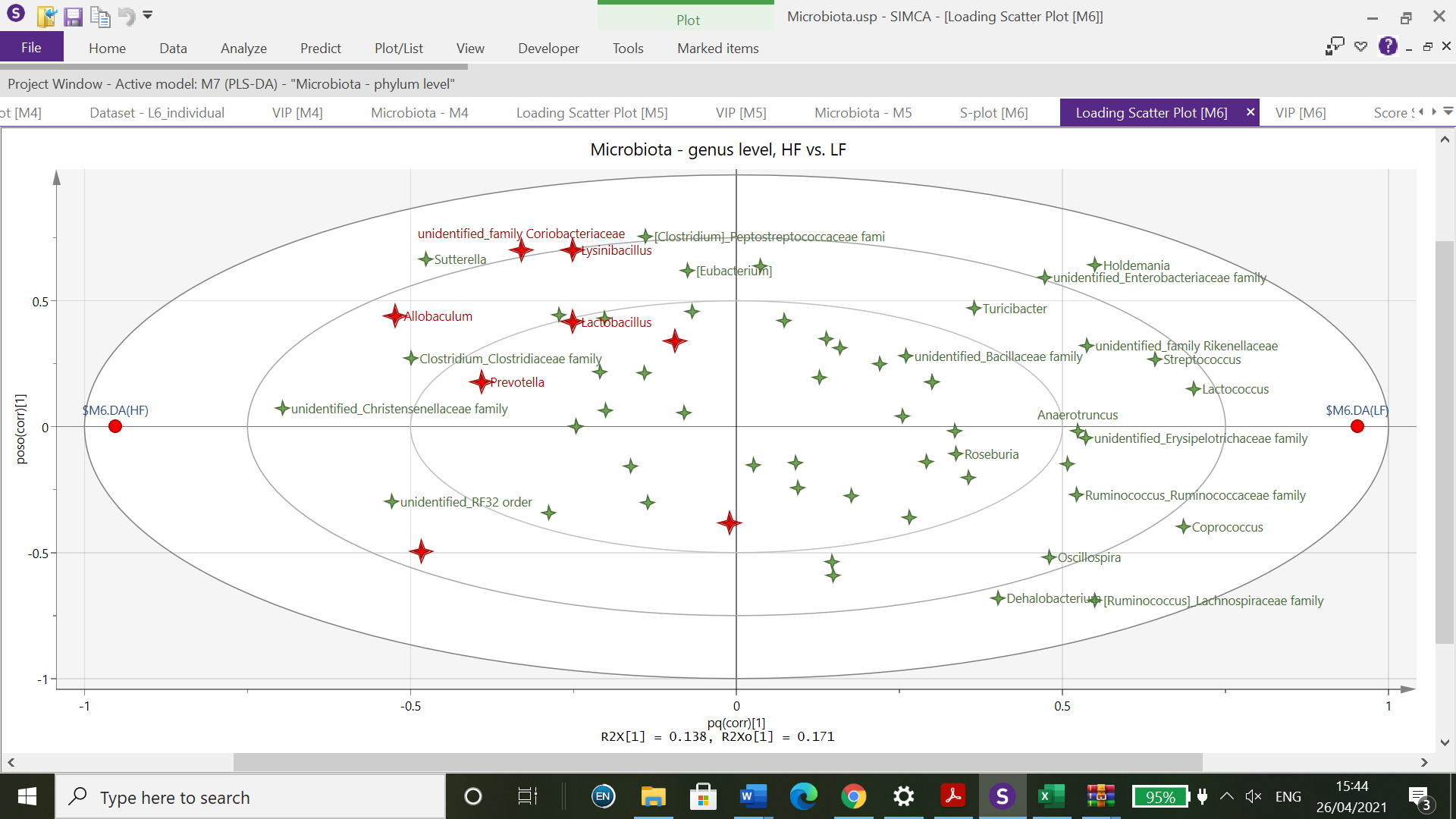


**Figure S1c.** Caecal microbiota at genus level in ApoE-/- rats fed high-fat (HF) or low-fat (LF) diets. The two groups are shown as circles, bacterial genera as 4-point stars. Red stars are bacterial genera that were higher in the ApoE-/- rats (HF) compared with conventional rats (N) fed the same high-fat diets.


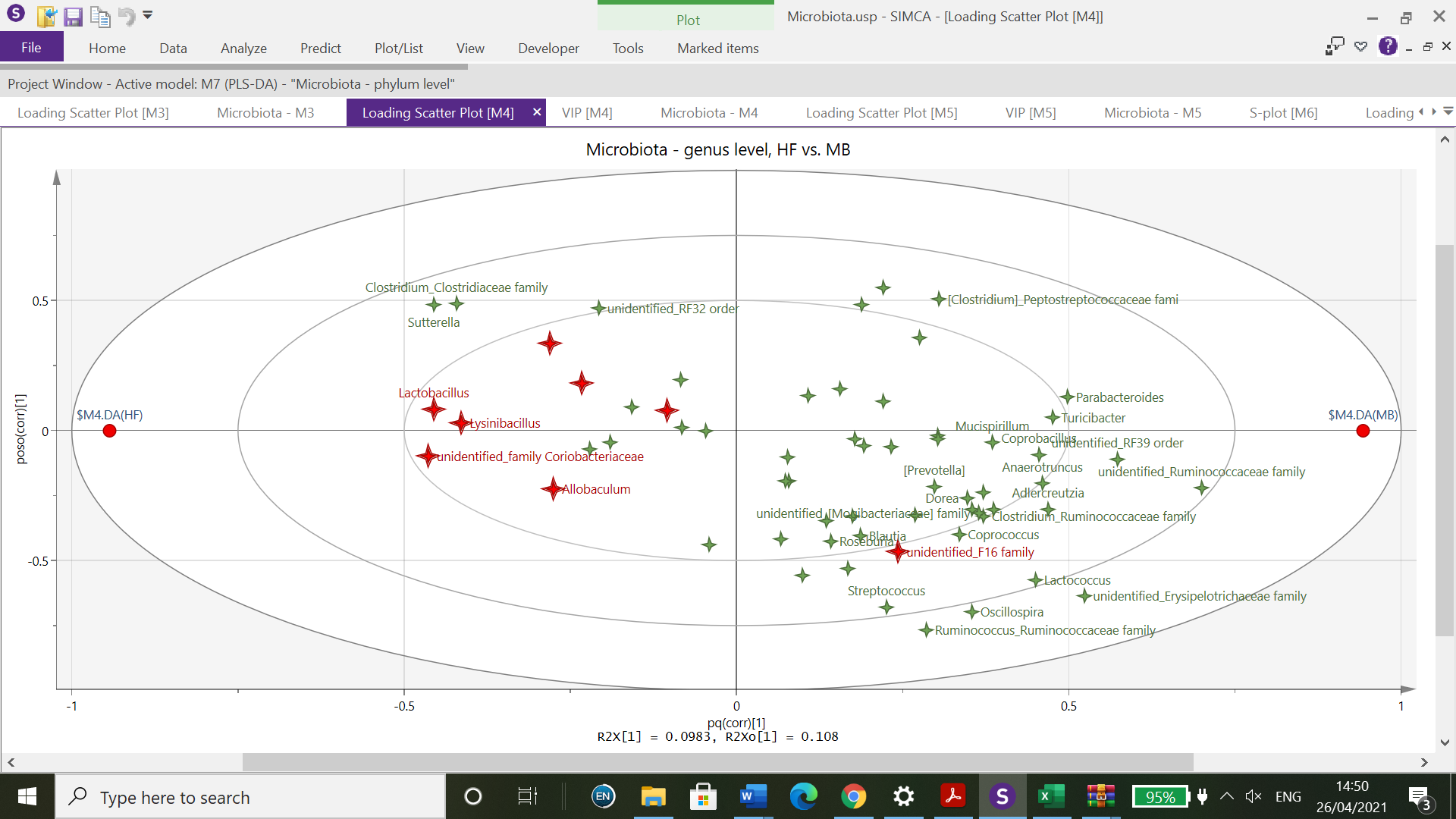


**Figure S1d.** Caecal microbiota at genus level in ApoE-/- rats fed a high-fat diet (HF), or the same diet supplemented with 1% monobutyrin (MB). The two groups are shown as circles, bacterial genera as 4-point stars. Red stars are bacterial genera that were higher in the ApoE-/- rats (HF) compared with conventional rats (N) fed the same high-fat diets.


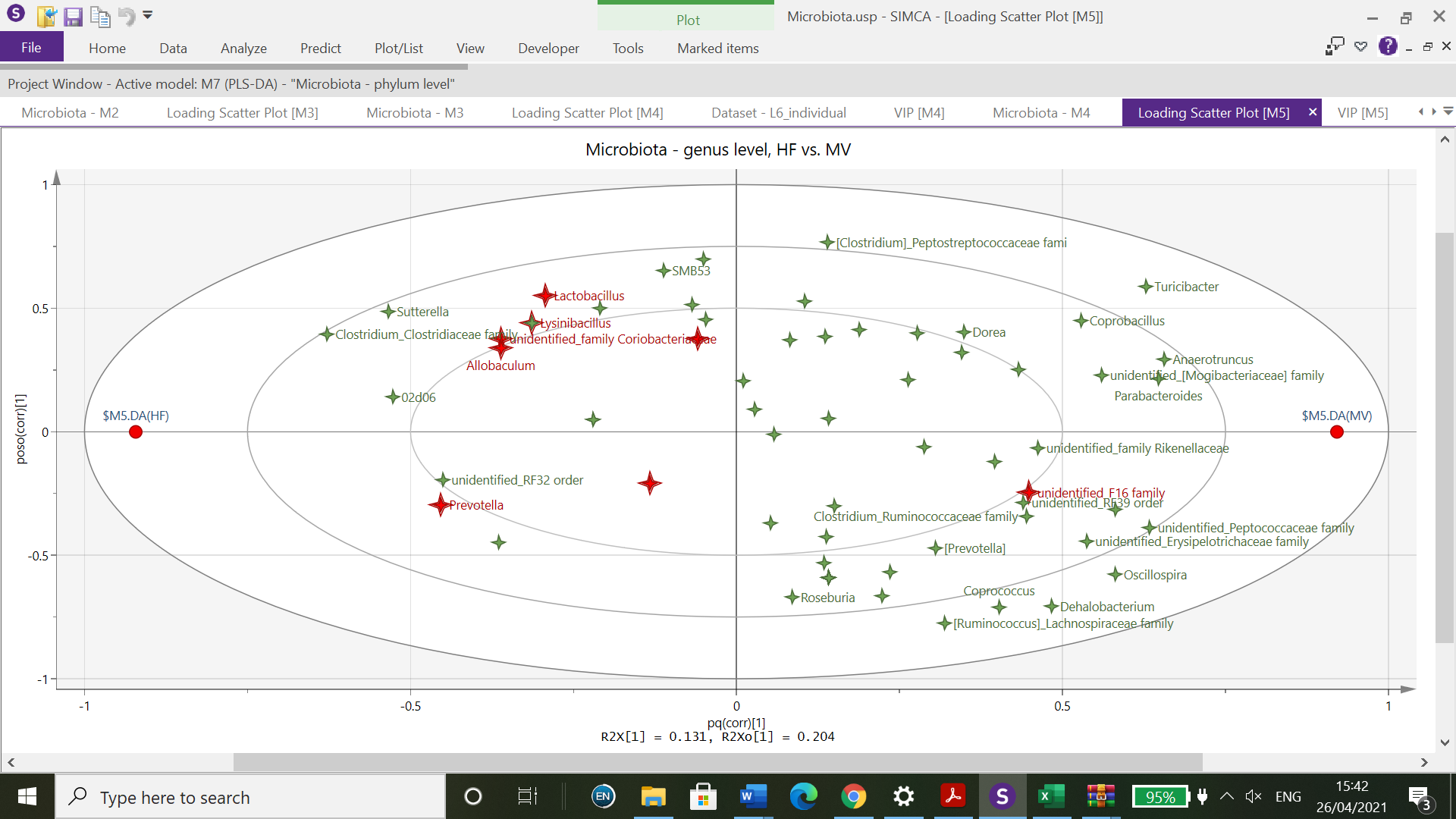


**Figure S1e.** Caecal microbiota at genus level in ApoE-/- rats fed a high-fat diet (HF) or the same diet with 1% monovalerin (MV). The two groups are shown as circles, bacterial genera as 4-point stars. Red stars are bacterial genera that were higher in the ApoE-/- rats (HF) compared with conventional rats (N) fed the same high-fat diets.

**Correlation of caecal microbiota with bile acids**

In the MB group (Figure 3a in the main manuscript), the amount of caecal CA was positively associated with *Bacillaceae* family (r=0.6487, p=0.05), *Lactobacillus* (r=0.6485, p=0.049), *Clostridium* in the *Clostridiaceae* family (r=0.6485, p=0.049), *Allobaculum* (r=0.7823, p=0.012), and *Holdemania* (r=0.6659, p=0.0434), but negatively correlated with *Coprococcus* (r=-0.6606, p=0.0438). Linking caecal CA to data previously analysed and published^2^, CA was also associated with serum SCFAs including total and individual concentrations of acetic, propionic, butyric and valeric acids (r= -0.76 to -0.90, p<0.0149 to p<0.0008), and gene expression of occludin in the brain (r=0.9429, p=0.0167). Caecal UDCA was positively correlated with *Bacillaceae* family (r=0.6786, p=0.0389), *Clostridia* class (r=0.6833, p=0.0444), *Clostridiaceae* family (r=0.6848, p=0.0347), *Clostridium* in the *Clostridiaceae* family (r=0.7333, p=0.0202), *SMB53* (r=0.6606, p=0.0438), *Epulopiscium* (r=0.7006, p=0.0222), *Peptostreptococcaceae* family (r=0.7128, p=0.0264), *Clostridium* in the *Ruminococcaceae* family (r=0.7006, p= 0.0222), *Mogibacteriaceae* family (r=0.7697, p=0.0126), *Tissierella*_*Soehngenia* (r=0.7006, p=0.0222) and *Holdemania* (r=0.8081, p=0.0083), as well as with propionic acid (r=0.7381, p=0.0458), and occludin (r=0.9429, p=0.0167) in the brain. UDCA was further negatively correlated with *Cyanobacteria* phylum and its *YS2* order (r=-0.7212, p=0.0234), *Clostridiales* order (r=-0.7697, p=0.0126), *Ruminococcus* in the *Lachnospiraceae* family (r=-0.648, p=0.049) and serum valeric acid (r=-0.8061, p=0.0072).

In the MV group (Figure 3b), UDCA was positively associated with *Lactobacillus* (r=0.6848, p=0.0347), *Turicibacter* (r=0.7988, p=0.0078), an unidentified genus (r=0.8667, p=0.0022) and *Clostridium* (r=0.6606, p=0.0438) in the *Clostridiaceae* family, *SMB53* (r=0.8788, p=0.0016), *Peptostreptococcaceae* family (r=0.8493, p=0.0032), *Coprobacillus* (r=0.6659, p=0.0434), and *Sutterella* (r=0.7599, p=0.0136), and negatively associated with *Prevotella* (r=-0.7006, p=0.0222), *Coprococcus* (r=-0.6748, p=0.037), *Ruminococcus* in the *Lachnospiraceae* family (r=-0.6485, p=0.049), and *Oscillospira* (r=-0.697, p=0.0306).

In the LF group, UDCA was positively associated with *Proteobacteria* phylum (r=0.7226, p=0.0221), *S24-7* family (r=0.0824, p=0.0075), *Lactococcus* (r=0.463, p=0.0484), *Streptococcus* (r=0.7744, p=0.0114), *Clostridiales* order (r=0.7122, p=0.0254), *Christensenellaceae* family (r=0.6951, p=0.0304), *Clostridium* in the *Clostridiaceae* family (r=0.6748, p=0.0372), *Blautia* (r=0.7599, p=0.0139), *Dorea* (r=0.75, p=0.0159), *rc4-4* (r=0.766, p=0.013), *Peptostreptococcaceae* family (r=0.6554, p=0.0454), *Ruminococcaceae* family (r=0.6565, p=0.0448), *Enterobacteriaceae* family (r=0.6626, p=0.0418), and negatively associated with *Verrucomicrobia* phylum and its genus *Akkermansia* (r=-0.7781, p=0109), *Bacillaceae* family (r=-0.8091, p=0074), and *Anaerotruncus* (r=-0.7809, p=0.0124).

In the HF group (Figure 3c), CA was correlated positively with the small intestinal expression of ZO-1 (r=0.7, p=0.0433). UDCA was only correlated with urinary mannitol (r=-0.693, p=0.0308) and not with any bacteria.

**References**

1. Klindworth, A.*, et al.* Evaluation of general 16S ribosomal RNA gene PCR primers for classical and next-generation sequencing-based diversity studies. *Nucleic Acids Res* **41**, e1 (2013).

2. Nguyen, T.D., Hallenius, F.F., Lin, X., Nyman, M. & Prykhodko, O. Monobutyrin and Monovalerin Affect Brain Short-Chain Fatty Acid Profiles and Tight-Junction Protein Expression in ApoE-Knockout Rats Fed High-Fat Diets. *Nutrients* **12**(2020).
